# Resolution of Complex Issues in Genome Regulation and Cancer Requires Non-Linear and Network-Based Thermodynamics

**DOI:** 10.3390/ijms21010240

**Published:** 2019-12-29

**Authors:** Jekaterina Erenpreisa, Alessandro Giuliani

**Affiliations:** 1 Cancer Research Division, Latvian Biomedicine Research and Study Centre, LV1067 Riga, Latvia; 2 Environmental and Health Department, Istituto Superiore di Sanità, 00161 Rome, Italy; alessandro.giuliani@iss.it

**Keywords:** genome positional information, transcription speciation, self-organisation, chromatin network, cancer resistance to drugs, reprogramming, cancer reversion therapy

## Abstract

The apparent lack of success in curing cancer that was evidenced in the last four decades of molecular medicine indicates the need for a global re-thinking both its nature and the biological approaches that we are taking in its solution. The reductionist, one gene/one protein method that has served us well until now, and that still dominates in biomedicine, requires complementation with a more systemic/holistic approach, to address the huge problem of cross-talk between more than 20,000 protein-coding genes, about 100,000 protein types, and the multiple layers of biological organization. In this perspective, the relationship between the chromatin network organization and gene expression regulation plays a fundamental role. The elucidation of such a relationship requires a non-linear thermodynamics approach to these biological systems. This change of perspective is a necessary step for developing successful ‘tumour-reversion’ therapeutic strategies.

## 1. Information Crisis in Bioscience and Cancer Research

The discovery of a linear cause/effect relation was a signature of the effective explanation since the very beginning of modern science. Everyone knows that any linear relation has limitations (e.g., the physical boundaries of the system) and only holds when particular requirements are met, but these limitations are too often overlooked, especially in biomedical research. Thus, the logic of the linear causality penetrated modern education and science with no caveats regarding the scale and boundary conditions.

The elucidation of DNA double helix and the breaking of genetic code consisting of three DNA nucleotide letters allowing for the transcription of genes for protein synthesis were two great discoveries of the 20th century. This breakthrough strengthened the reductionist trend and favoured the development of genetic engineering up to CRISPR technology in the 21st century. In this case, linearity is embedded in a linear deterministic flow of information while considering each gene product as an autonomous agent in charge of a specific physiological role. However, until now, this approach has not provided an answer to a simple question in spite of evident technological success: how the same genome with the same genes differentially regulates their expression in different tissues of multicellular organisms. As wittily said by Carl Woese [1] “an engineering biology might still show us how to get there; it just does not know where “there” is’’. The lacking “there” is a constitutive problem of modern biology.

The fact that cancer problem is more complex than we have thought and needs re-thinking was recently recognised by leaders in cancer research Robert Weinberg [2] and Douglas Hanahan [3] after the failure of cancer genome sequencing projects to support a somatic mutation theory of cancer. The latter, in turn, largely makes a current base of the costly “precision medicine”, which is also beginning to frustrate the hopes: “The targeting of lower-level agents (genes and pathways) provides unsatisfactory results at higher levels of this system such as clinical outcomes” [4]. In addition, the reproducibility crisis has been claimed to comprise 75% of the published reports in biomedicine, with 95% in cancer research [5,6,7].

The general situation appears as an information crisis in bioscience and biomedicine. While the nature of the crisis is multi-faceted and it goes from strictly scientific issues (the lack of the ability to put into context the relations between different layers of biological organization) to research policy asking for rapid solutions and, thus, not financing very basic research approaches that shake the boundaries of accepted dogma.

Here, we propose a few considerations regarding the origin of both problems, together with some theoretical hints to overcome the crisis.

## 2. Regulation of the Human Genome: Networking by Self-Organisation Is the Second Principle of Genome Regulation after WATSON-CRICK Complementarity

**Figure 1:**
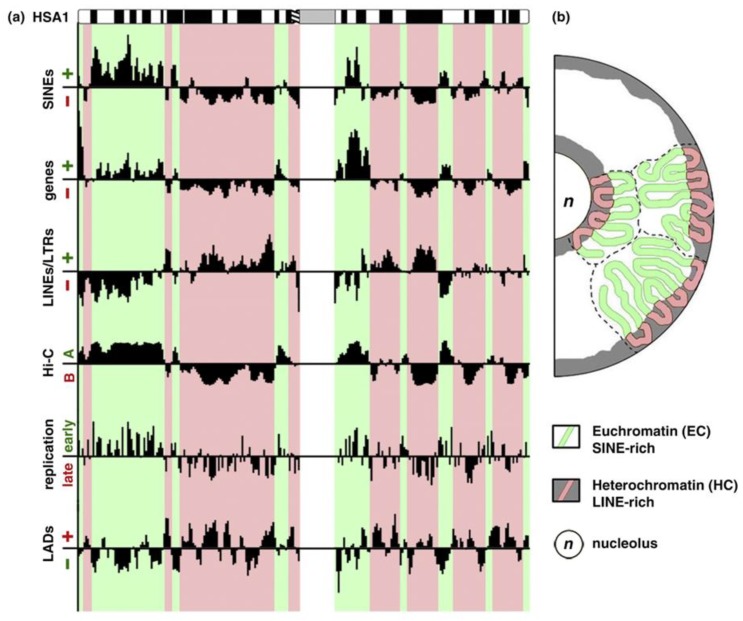
Euchromatic and heterochromatic chromosome regions and their spatial separation in the nucleus. (**a**) Euchromatin (EC) and heterochromatin (HC) domains revealed by different approaches showing the principal components and differential replication timing. (**b**) EC resides in the nuclear interior, whereas HC localizes to the nuclear and nucleolar periphery. A scheme is republished from [14], Figure 1, with the licence provided by Elsevier and Copyright Centre.

In mammals, only 2% of the genome DNA is coding the translated proteins. Approximately 50% of DNA is composed of simple reiterated sequences enclosing; in addition, a considerable portion (45%) of mostly silent transposable elements in the human genome. The clustering of heterochromatin (constitutive and facultative) is differently patterned in relation to the radial gradient between the nucleolus and nuclear envelope in the cells of different tissues [8,9,10,11,12,13,14,15,16], as in Figure 1; this pattern is altered in cancer, as it is known to all pathologists.

In the past, several scientists suggested that heterochromatin regulates the differential expression of genes by biophysical mechanisms, by a force field gradient acting in the nucleus space [17,18,19,20,21]. Currently, there are enough results that include those coming from the nucleus image analysis and chromosome conformation capture techniques, which suggests that the heterochromatin mediating the gene silencing position effect is responsible for the differentiation-specific expression of the genetically active euchromatin [22,23,24,25,26,27,28,29,30]. The above evidence suggests that a three-dimensional heterochromatin (3D) topology created by 3D contacts represents a lacking “there”, i.e., the positional information of the supra-chromosomal network in the cell nucleus for transcription speciation. This positional information is specified by the loops of the ~ 1Mbp topology-associated chromatin domains (TADs) [31]; these loops join gene enhancers with promoters that are are insulated from neighbour TADs, and can converge to mRNA transcription hubs, likely uniting the genes for specific functions [24,32,33,34].

The integrity of the chromatin network is reciprocally dependent on ongoing transcription [35,36]. This implies that this spatial system should change with transcription speciation by differentiation in normal development or by induction [37]. Understanding how positional information is translated into functional one is crucial in overcoming the above-mentioned knowledge crisis, because it gives the ‘missing link’ between the specificity of transcription and chromosomal organization.

## 3. The Genome “Maps” of Positional Information Need Phase Transitions

From a purely structural point of view, the position of a system becomes informative if (and only if) it introduces a symmetry break in the space. The term ‘symmetry break’ is coming from statistical mechanics that can be interpreted as going from a situation in which a system can choose between different equally probable and, thus, symmetric, positions to a situation in which one choice becomes much more probable than the others and the system is channelled toward the corresponding direction.

In an abstract sense, the genome would be a ‘homogeneous space’ if a 2 m-long DNA molecule is evenly (symmetrically) distributed in the 10 µm-sized nucleus. The readout of any gene would be equally probable. It is not the case as the negatively charged DNA is many-fold packed into the chromatin by specific positively charged proteins (histones). Moreover, through contacts of the distant genome parts and self-organisation of the euchromatin and heterochromatin domains this DNA folding is topologically uneven, thus undergoing symmetry break. This can create positional information for the differential tissue-specific transcription of the genes, either exposed in loose euchromatin or hidden in densely packed heterochromatin. In this sense, the establishment of positional information needs symmetry break. Symmetry break in the genome space means that its space becomes a “map” with recognizable coordinates for the whole genome forming a network [38,39,40] of wired contacts that impose a topological grid (and subsequently a specific address) on nuclear space. Symmetry breaking causes physical phase transitions that operate by hydration, change of charge, aggregating, and crowding of the genetic material. Physical phase transition is also a prerequisite of self-organisation [41].

Therefore, we can safely state that networking by self-organisation is the second principle of genome regulation after Watson-Crick complementarity. This is the ‘missing link’ that, when fully investigated, could answer Carl Woese’s question [1].

The idea of self-organisation in biology has a long history [39], but it only entered nucleome studies in the 21st century [42,43,44] that were strengthened by the Hi-C genome methodology [45,46]. In parallel, phase transition by self-organized criticality (SOC) was revealed in quantitative studies of transcriptome dynamics during cell fate change, such as an oocyte-to-embryo transition in early embryogenesis and induction of the commitment for cell differentiation [47,48].

Each cell type corresponds to a specific configuration in a genome map. It is mandatory to destroy the previous order for creating a new network to change cell fate [47,49] and, subsequently, a new map of positional information. A system that is able to perform such a task should possess the dissipative features (to be both open and energy-saving) that are necessary for sustaining the onset of new ordered structures by dissipating the energy excess [39,49,50].

The toy-model of self-organization by criticality (SOC) is a sand-pile to which we pour new sand, a grain at the time [51]. This system attains a ‘stable critical state’: when the slope becomes too steep, somewhere on the pile, the grains slide down, causing a small avalanche. These small fluctuations do not alter the system (sand-pile slope): the added sand balances the continuous avalanches and the shape of the pile remains the same. Nevertheless, occasionally, an added grain can cause a large catastrophic avalanche by a domino effect that involves progressive smaller avalanches falling down until the base and, thus, flattening the entire sand-pile (long-range correlation, which is typical of transition states). The small perturbations that are caused by added grains are the counterpart of the continuous solicitations that are experienced by a biological system from its microenvironment and its stability comes from the dissipation of such perturbations by small fluctuations around the ‘native state’.

A network structure sustains the ‘domino-effect’ that is necessary for spreading the signal and, at odds with real sand-piles, where the onset of ‘catastrophic avalanches’ happens by chance and with no peculiar location, a ‘purposely wired’ network is able to discriminate the non-relevant and informative perturbations in a way that is analogous to the allosteric effect in protein molecules [52,53]. This translates into the ‘response specificity’ of biological systems, in which only certain stimuli (independently of their relative energy) are able to elicit a relevant response [52]. The allosteric effect of an enzyme is the most studied case of ‘non-local’ effect mediated by the wiring protein structure (contacts among residues generated by folding), where not only the local active site is important, but also conformational changes of the entire structure of the protein molecule that impinges on the functionality of the active site [53]. Similarly, the preferential way of the chromatin networking might be determined by the anisotropic (symmetry breaking) super-packaging of heterochromatin [54], with its aggregation-stimulating capacity to impinge ‘a sticky silence’ on the large genome domains [15,55]. The non-coding RNA that is transcribed from heterochromatin [56] can also electrostatically contribute to its packaging [25,57]. The spatial compartmentalization of the nucleus is maintained by relatively simple basic physicochemical principles, including electrostatic and hydrophobic forces that generate an extremely detailed ‘nuclear topology’ giving a material basis to robust gene expression regulation, as indicated by Ronald Hancock [58]. However, the latest studies on single cells revealed that, at a level of TAD loops, the genome organization is heterogeneous and transcription itself might shape and stabilize the TAD-shaping contacts [59,60]. These data show the complexity of the system, both being consistent with the SOC features described above and with the principles of reciprocal causation [61].

## 4. Differential DNA Replication Timing Translates Temporal Information into Positional Information

The discrimination between relevant and non-relevant signals asks for positional information and clearly includes the fourth dimension: time. The positional information in the cell nucleus is, in fact, set by differential replication timing, early for transcriptionally active and late, for inactive genome parts [62], see also Figure 1a. The simultaneously replicating chromatin domains become both similarly epigenetically marked and spatially joined [63,64,65,66]. This replication timing, in turn, correlates with the speed of rapid oscillatory motions of the corresponding TADs [67], which were first identified through the motion of replication clusters [68]. We can safely say that, the temporal information is translated into spatial symmetry breaking for differential transcription operating in G1-phase through the mechanism of differential replication timing coupled with the epigenetic marking of the chromatin. This implies that a significant spatial re-arrangement of the nuclear configuration changing the genome transcription profile can be epigenetically transmitted along with subsequent cell divisions [69]. It is evident that we are facing a sort of a ‘second genetic code’ operating with a four-dimensional (4D)-language (and thus much more difficult to interpret with respect to the mono-dimensional logic of genetic code based on Watson-Crick pairing along a linear sequence). This spatio-temporal language is dealing with self-similar (fractal) folded structures across different organization layers due to the need of 10,000-fold packaging of the 2m-long DNA thread in a cell nucleus [70,71]. An interesting approach for their study in individual cells is correlative microscopy with increasing resolution [72].

The onset of a state transition (and the subsequent state change that in the case of the nucleus corresponds to the reorganization of chromatin network) represents a ‘giant fluctuation’ that invades the entire network [73,74,75]. Thus, the supra-chromosomal network structure should be also arranged in a manner that provides the necessary elasticity and order parameters that correlate with the fluctuation amplitudes [38,76]. Oscillations of the chromatin networks have been registered. They likely start with the pulsation of the clusters of nucleolus-associated heterochromatin [77] that involve the nucleoli (whose pulsations were already known to Balbiani (1883), as cited by [78,79], and end up into overall transcriptional bursting in individual cells [80,81,82] enabling regulatory information to be coordinated and transmitted in a digital manner [83]. These events increase the coherence and synchronization of the whole cell population, as revealed in different stress conditions [84,85,86]. The oscillations for orchestration of cellular coherence have ultradian periodicity (4min–4h) [87]. Currently, the genome oscillations are in the focus of 4D nucleome studies [25,88,89].

## 5. Deterministic Chaos for Cell Fate Change: Inevitable Heterogeneity and Fluctuations

The enucleation of bifurcation space points is another crucial issue to consider, where a system undergoes a shift from one state to another. The tissue-specific differentiation states are relatively few, only ~250 human tissues and ~440 cell types per 22,000 protein-coding genes, whose compositions (in the case of lack of any preferred global configuration of the entire genome organization) would have been over billions [90]. This means that these extremely rare and robust compositions of active/inactive genes (attractors) cannot be set by the laws of linear thermodynamics. It is non-linear thermodynamics instead, which can help to set these low probable (in the case of a purely random choice of gene expression levels) events. Within its action, a rare attractor can be found by the random search of the fluctuating system in a multi-dimensional space of the possibilities, which involves the entire cell community in feed-back interactions with permissive environment; however, the system becomes “hooked” (determined) when an appropriate sensor/inducer can give rise and forward a channelled trajectory toward it [91]. This type of behaviour of dynamic systems, which “harness stochasticity” and allow for the creative choice, was first recognized by Edward Lorenz and termed as “deterministic chaos” [92], which becomes “tamed” (set at the lowest energy state) in the appropriate attractor [93,94,95,96].

From the above considerations, we see that any cellular system changing its fate, when in search of the appropriate differentiation attractor (this first step is designated in biology as a commitment), should be both heterogeneous and fluctuating. It also means that to channel the process further, initially a few outliers, a small avant-garde group might be involved, which can then self-organize the whole cell population [97], where chaotic ‘white noise’ of a strongly fluctuating system might be paradoxically favourable [98].

For an experimentalist, this translates into the need, in order to catch the ongoing dynamical process, to study individual cells (microscopy, in situ and flow cytometry, single-cell transcriptome sequencing), and to consider not only the population averages, but also population heterogeneity. As analysed by [82], the relationships between inputs to outputs can either be deterministically specified, diversity generating, or buffering. It also means that the traditional methods of studying the DNA, RNA, and proteins from the population extracts may be not informative enough, at some point even misleading, as showing no statistically significant changes, where only the initiating small groups of cells may be already starting to channel the process. This implies that we should not neglect the statistical outliers which may be the forerunners of the change [98]. Therefore, the reproducibility crisis in biomedicine might be associated not so much with the incorrect measurements and statistical errors, as with the still not recognized dissipative nature of their objects.

## 6. Cancer Cell Treatment Resistance Is Ensured by Deterministic Chaos and Reprogramming to the Embryonic State

In particular, the non-equilibrium thermodynamics is related to the nature of cancer cells and their resistance to the conventional radio-chemotherapy treatments that are applied in oncology clinics, which aim to kill these cells. The majority is killed, while a small minority (in some cases only 0.01% or even less) inevitably (at least, in the case of *tp53* mutants) escapes death and finds a new attractor while using the regulations of the tamed chaos [96,98]. This strict minority of cells can give rise to cancer relapse. The “statistically irrelevant” behaviour of cancer drug escapers is due to the change of their very nature—becoming reprogrammed to the state of an egg, embryo, or adult stem cell, thus acquiring the toti- or pluri-potency [99,100,101,102,103,104,105,106,107,108,109,110,111,112,113]. The capability of deep reprogramming particularly depends on the *tp53* (tumour suppressor) insufficiency [114], which is common in the most aggressive cancers [115].

The cells reaching the state equivalent to the embryonal stem cell (ESC) should destroy the positional information of the normal tissue of origin. ESC themselves display a highly dynamic loosely bound architectural chromatin proteins of the less constrained (than in differentiated cells) cell nucleus [116]. They also set bi-valent chromatin domains (as detected by activating and repressing histone marks at the promoters of developmental genes), which enable transcription fluctuations from repressed to active to occur [117]. Thus, the system can be channelled toward different “predetermined” pathways/attractors. Moreover, some opposing pathways (e.g., proliferation versus death, senescence versus oncogenesis) can be driven, even by the same pleiotropic genes, f.ex., by *c-myc* and *ras* [118,119], or by reversible shifts of *pou5f1* from full to spliced transcript [120]. In the emergency conditions of the limited resources, this tactic of coupling the opposite effects driven by the same genes, even within the same bi-potential cell undergoing asymmetric division, allows for concentrate growth factors for a few channelled survivors [91,121].

This is not strange, given that any complex system interprets the incoming signals according to its state [122,123].

This inescapable ‘system state dependence’ of any observed feature should push toward a general re-thinking of many non-critically accepted statements, like the widely believed tenet that high proliferation speed is the principal marker of cancer cells.

As a matter of fact, the main feature of cancer cells is not their speed of proliferation (the embryonal cells can proliferate faster), but their capability to be reprogrammed to the totipotent or pluripotent profile. Just on the contrary, the reprogramming that is induced by irradiation or anti-cancer drugs is associated with uncoupling from cell division and transient drop of proliferation, leading to tetraploidy and multi-nucleation [112,124,125,126], and just those cells serve (after de-polyploidisation and return to mitoses) for the escape from damage and cancer relapse [127,128]. The whole-genome doubling of the reprogrammed cell, in turn, can allow for the creation and bifurcation of the two different epigenomes for asymmetric cell division of two daughters [121,129].

Hence, these reprogrammed polyploidised cancer cells acquire high genome expression plasticity (the capability to visit and set in different attractors), allowing for acquiring an otherwise improbable resistance to extinction, which is the essential hallmark of cancer [103,106,108,130]. The process also involves accelerated cell senescence—the metastable bi-potential state, which appears to be “another face” of self-renewal associated with polyploidy [131,132,133,134,135].

## 7. Cancer Cells Recapitulate the Stress-Adaptive Programs of Unicellulars and Early Metazoans

This extreme endurance of tumour cells is likely borrowed from the phylogenetic evolution of cell response to stress [136,137], where the elastic epigenetic reprogramming was the main tool in the search of the available survival pathways. In this reprogramming, the protozoan, and even prokaryotic transcription cassettes (in the latter, first of all, of the DNA repair pathways), become dominating in the transcriptome of cancer gene network [138,139,140,141,142], particularly in association with polyploidy [143,144]. Accordingly, cancer is not only reprogrammed to the expression state of a gamete, a two-cell embryo or morula of a multicellular organism [100,113], but it can recapitulate through reprogramming the adaptive pathways of unicellular organisms. Those developed on Earth through the 3.5 billion-year history, which have survived six Earth-wide catastrophes, some destroying up to 75% of species.

In turn, the early embryonal development in mammals also bears the phylogenetic features of the transition from unicellular to multicellular organisms. This transition evolved little new genes and it was mostly operating with multi-nuclearity, transient colonial forms, ploidy cycles [145,146], chromatin diminution [147], facultative sex [148], and asexual reproduction [149,150]. Interestingly, the earliest tumours were discovered within the same evolutionary forms, in basic metazoan *Hydra*, the authors concluded: “cancer is as old as multicellular life on Earth” [151]. Unicellular-to-multicellular transition, as better studied on cellular slime moulds going from unicellular-to-multicellular transition and back in each life cycle, is mostly based on epigenetic plasticity and self-organisation [152].

## 8. Chaos in Cancer is Akin to Chaos in an Early Embryo Which Serves to Change Cell Fate

Moreover, the impressive chaos was found in the genome of an early mammalian embryo (up to blastocyst stage), in striking similarity with cancer. Chromosome instability, aberrant mitoses, heteroploidy, anaphase bridges, structural chromosome aberrations, and loss of heterozygosity in some single cells, etc. manifest it [153,154]. During the oocyte-to-embryo change, a critical phase transition occurs in transcription involving thousands of herein low expressed genes, as determined by Tsuchiya et al. [48], while Peaston and colleagues [155] found the activation of the thousands of previously silent endogenous retroelements at that same time. They suggested that massive activation of retroviruses (released from their heterochromatic shelters by genome-wide demethylation) provides potential scope for large-scale coordinated epigenetic fluctuations that can trigger this cell fate change, thus potentially driving it by the rules of the non-equilibrium thermodynamics. The hyper-dynamic behaviour of the structural chromatin proteins, with the diffusion of chromocentres found in ESC [116] (directly indicating the erasure of positional information) corresponds to the same type of regulation. In other words, albeit shocking a median biologist, chaos in early embryo paradoxically serves cell fate determination [156].

## 9. Reprogramming of Positional Information Can Be Used for Cancer Reversion Therapy

By the same rules of the non-linear thermodynamics and, as mentioned above, the aggressive radio-chemotherapy inducing reprogramming associated with reversible polyploidy and genome instability, paradoxically favours the opposite to the aimed effect—creation, selection, and survival of residual resistant clones and, therefore, is generally without perspective [106,113,128]. The exit from the circulus vicious of cancer treatments might be searched either in interruption of reprogramming (that might be difficult or even impossible) or in the opposite direction, while using the toti-pluripotent reprogrammed state of cancer cells for their channelled normalisation (differentiation, which epigenetically can take over the mutations [157]). For this purpose, the appropriate morphogenic embryonal inducers, regeneration fields [158], and the structured 3D environment—putting tumours in context [159] and changing the chromatin folding—can be used [160]. This approach can, at least, stop the invasion and metastatic spread of cancer, which is the main cause of patient mortality.

In turn, if we consider the perspective of cancer normalisation by differentiation [161,162,163], the open problem of the whole genome regulation by topological mechanisms for tissue differentiation should be solved in parallel. These two are the converging issues that need to consider the non-linear thermodynamics of the living matter, which ought to become the mainstream approach in the very near future.

Up to now, these systemic approaches are still in their infancy for the therapeutic application, although the theoretical background of cell fate reversion is already established by many different studies comprising a coherent picture [50,164,165,166]. During the last two decades, it has been firmly ascertained that adult somatic cells—either normal or pathologic—can be efficiently “reprogrammed”, recovering the status of Induced Pluripotent Stem Cells (iPSCs) and then redirected to acquire a differentiated phenotype [167]. Still more intriguing is the action of retinoic acid, which was initially demonstrated to promote reversion of teratocarcinoma [168], has been later recognized to induce a nearly complete differentiation of leukemogenic cells in acute promyelocytic leukemia through the induction of terminal differentiation into granulocytes [169], thus reversing cell fate from cancer to healthy fully differentiated state, which can, in turn, lead to partial or complete clinical remission. Recently [170], such a cell fate reversion was also observed for a solid tumour. The most recent achievement, the conversion of breast invasive cancer cells into adipocytes, was shown in a mouse model while using PPAR-gamma (member of nuclear receptors regulating adipocyte differentiation) agonist combined with the MEK inhibitor [171].

The oncoming future will eventually verify the 1948 prediction that was made by Warren Weaver (one of the fathers of mathematical information science), who posited that the study of networked systems represents the only possible way to cope with the complexity of life [172].

## References

[1] Woese C.R. (2004). A new biology for a new century. Microbiol. Mol. Biol. Rev..

[2] Weinberg R.A. (2014). Coming full circle-from endless complexity to simplicity and back again. Cell.

[3] Hanahan D. (2014). Rethinking the war on cancer. Lancet.

[4] Heng H.H., Liu G., Alemara S., Regan S., Armstrong Z., Ye C.J., Sturmberg J. (2019). The Mechanisms of How Genomic Heterogeneity Impacts Bio-Emergent Properties: The Challenges for Precision Medicine. Embracing Complexity in Health.

[5] Ioannidis J.P.A. (2005). Why most published research findings are false. PLoS Med..

[6] Nuzzo R. (2014). Scientific method: Statistical errors. Nature.

[7] Voosen P. (2015). Amid a Sea of False Findings, the NIH Tries Reform. Chron. High. Educ..

[8] Bartova E. (2002). Nuclear structure and gene activity in human differentiated cells. J. Struct. Biol..

[9] Beil M., Dürschmied D., Paschke S., Schreiner B., Nolte U., Bruel A., Irinopoulou T. (2002). Spatial distribution patterns of interphase centromeres during retinoic acid-induced differentiation of promyelocytic leukemia cells. Cytometry.

[10] Kinney N.A., Onufriev A.V., Sharakhov I.V. (2015). Quantified effects of chromosome-nuclear envelope attachments on 3D organization of chromosomes. Nucleus.

[11] Mayer R., Brero A., von Hase J., Schroeder T., Cremer T., Dietzel S. (2005). Common themes and cell type specific variations of higher order chromatin arrangements in the mouse. BMC Cell Biol..

[12] Németh A., Längst G. (2011). Genome organization in and around the nucleolus. Trends Genet..

[13] Padeken J., Zeller P., Gasser S.M. (2015). Repeat DNA in genome organization and stability. Curr. Opin. Genet. Dev..

[14] Solovei I., Thanisch K., Feodorova Y. (2016). How to rule the nucleus: Divide et impera. Curr. Opin. Cell Biol..

[15] Van Steensel B., Belmont A.S. (2017). Lamina-Associated Domains: Links with Chromosome Architecture, Heterochromatin, and Gene Repression. Cell.

[16] Weierich C., Brero A., Stein S., von Hase J., Cremer C., Cremer T., Solovei I. (2003). Three-dimensional arrangements of centromeres and telomeres in nuclei of human and murine lymphocytes. Chromosom. Res..

[17] Lima-de-Faria A. (1954). Chromosome gradient and chromosome field in Agapanthus. Chromosoma.

[18] Lima-de-Faria A. (1980). Classification of genes, rearrangements and chromosomes according to the chromosome field. Hereditas.

[19] Nagl W. (1982). Condensed Chromatin: Species-Specificity, Tissue-Specificity, and Cell Cycle-Specificity, as Monitored by Scanning Cytometry. Cell Growth.

[20] Nagl W., Popp F.A. (1983). A physical (electromagnetic) model of differentiation. 1. Basic considerations. Cytobios.

[21] Popp F.A., Nagl W. (1983). A physical (electromagnetic) model of differentiation. 2. Applications and examples. Cytobios.

[22] Albiez H., Cremer M., Tiberi C., Vecchio L., Schermelleh L., Dittrich S., Küpper K., Joffe B., Thormeyer T., von Hase J. (2006). Chromatin domains and the interchromatin compartment form structurally defined and functionally interacting nuclear networks. Chromosom. Res..

[23] Cabianca D.S., Gasser S.M. (2016). Spatial segregation of heterochromatin: Uncovering functionality in a multicellular organism. Nucleus.

[24] Cremer T., Cremer C. (2001). Chromosome territories, nuclear architecture and gene regulation in mammalian cells. Nat. Rev. Genet..

[25] Cremer T., Cremer M., Hübner B., Strickfaden H., Smeets D., Popken J., Sterr M., Markaki Y., Rippe K., Cremer C. (2015). The 4D nucleome: Evidence for a dynamic nuclear landscape based on co-aligned active and inactive nuclear compartments. FEBS Lett..

[26] Erenpreisa J., Zhukotsky A., Kozlov A. (1993). The chromatin network: Image analysis of differentiating chick embryo chondrocytes. Eur. J. Histochem..

[27] Ērenpreisa J., Zhukotsky A. (1993). Interphase genome as the active space: Chromatin dynamics during chick embryo chondrogenesis. Mech. Ageing Dev..

[28] Kosak S.T., Groudine M. (2004). Form follows function: The genomic organization of cellular differentiation. Genes Dev..

[29] Pombo A., Dillon N. (2015). Three-dimensional genome architecture: Players and mechanisms. Nat. Rev. Mol. Cell Biol..

[30] Wiblin A.E., Cui W., Clark A.J., Bickmore W.A. (2005). Distinctive nuclear organisation of centromeres and regions involved in pluripotency in human embryonic stem cells. J. Cell Sci..

[31] Dixon J.R., Selvaraj S., Yue F., Kim A., Li Y., Shen Y., Hu M., Liu J.S., Ren B. (2012). Topological domains in mammalian genomes identified by analysis of chromatin interactions. Nature.

[32] Gonzalez-Sandoval A., Gasser S.M. (2016). On TADs and LADs: Spatial Control Over Gene Expression. Trends Genet..

[33] Sandhu K.S., Li G., Poh H.M., Quek Y.L.K., Sia Y.Y., Peh S.Q., Mulawadi F.H., Lim J., Sikic M., Menghi F. (2012). Large-scale functional organization of long-range chromatin interaction networks. Cell Rep..

[34] Spector D.L. (2001). Nuclear domains. J. Cell Sci..

[35] Haaf T., Ward D.C. (1996). Inhibition of RNA polymerase II transcription causes chromatin decondensation, loss of nucleolar structure, and dispersion of chromosomal domains. Exp. Cell Res..

[36] Papamichos-Chronakis M., Peterson C.L. (2013). Chromatin and the genome integrity network. Nat. Rev. Genet..

[37] Rajapakse I., Perlman M.D., Scalzo D., Kooperberg C., Groudine M., Kosak S.T. (2009). The emergence of lineage-specific chromosomal topologies from coordinate gene regulation. Proc. Natl. Acad. Sci. USA.

[38] Csermely P., Korcsmáros T., Kiss H.J.M., London G., Nussinov R. (2013). Structure and dynamics of molecular networks: A novel paradigm of drug discovery: A comprehensive review. Pharm. Ther..

[39] Karsenti E. (2008). Self-organization in cell biology: A brief history. Nat. Rev. Mol. Cell Biol..

[40] Soofi E.S. (1994). Capturing the Intangible Concept of Information. J. Am. Stat. Assoc..

[41] Landau L.D. (1937). On the theory of phase transition. Zh Eksp Teor Fiz.

[42] Cavalli G., Misteli T. (2013). Functional implications of genome topology. Nat. Struct. Mol. Biol..

[43] Misteli T. (2001). The concept of self-organization in cellular architecture. J. Cell Biol..

[44] Misteli T. (2007). Beyond the Sequence: Cellular Organization of Genome Function. Cell.

[45] Lieberman-Aiden E., van Berkum N.L., Williams L., Imakaev M., Ragoczy T., Telling A., Amit I., Lajoie B.R., Sabo P.J., Dorschner M.O. (2009). Comprehensive mapping of long-range interactions reveals folding principles of the human genome. Science.

[46] Rao S.S.P., Huntley M.H., Durand N.C., Stamenova E.K., Bochkov I.D., Robinson J.T., Sanborn A.L., Machol I., Omer A.D., Lander E.S. (2014). A 3D Map of the Human Genome at Kilobase Resolution Reveals Principles of Chromatin Looping. Cell.

[47] Giuliani A., Tsuchiya M., Yoshikawa K. (2017). Self-Organization of Genome Expression from Embryo to Terminal Cell Fate: Single-Cell Statistical Mechanics of Biological Regulation. Entropy.

[48] Tsuchiya M., Giuliani A., Hashimoto M., Erenpreisa J., Yoshikawa K. (2016). Self-Organizing Global Gene Expression Regulated through Criticality: Mechanism of the Cell-Fate Change. PLoS ONE.

[49] Kauffman S. (1971). Chapter 5 Gene Regulation Networks: A Theory for Their Global Structure and Behaviors. Curr. Top. Dev. Biol..

[50] Huang S., Kauffman S.A. (2012). Complex gene regulatory networks–from Structure to Biological Observables: Cell fate determination gene regulation. Comput. Complex..

[51] Bak P., Chen K. (1991). Self-Organized Criticality. Sci. Am..

[52] Nussinov R., Tsai C.-J., Csermely P. (2011). Allo-network drugs: Harnessing allostery in cellular networks. Trends Pharm. Sci..

[53] Paola L.D., Di Paola L., Giuliani A. (2015). Protein contact network topology: A natural language for allostery. Curr. Opin. Struct. Biol..

[54] Erenpreisa J. (1989). Two mechanisms of chromatin compaction. Acta Histochem..

[55] Marcand S., Gasser S.M., Gilson E. (1996). Chromatin: A sticky silence. Curr. Biol..

[56] Johnson W.L., Straight A.F. (2017). RNA-mediated regulation of heterochromatin. Curr. Opin. Cell Biol..

[57] Erenpreisa J., Krigerts J., Salmina K., Selga T., Sorokins H., Freivalds T. (2018). Differential staining of peripheral nuclear chromatin with Acridine orange implies an A-form epichromatin conformation of the DNA. Nucleus.

[58] Hancock R. (2014). The Crowded Nucleus. International Review of Cell and Molecular Biology.

[59] Finn E.H., Pegoraro G., Brandão H.B., Valton A.-L., Oomen M.E., Dekker J., Mirny L., Misteli T. (2019). Extensive Heterogeneity and Intrinsic Variation in Spatial Genome Organization. Cell.

[60] Finn E.H., Misteli T. (2019). A genome disconnect. Nat. Genet..

[61] Svensson E.I. (2018). On Reciprocal Causation in the Evolutionary Process. Evol. Biol..

[62] Rhind N., Gilbert D.M. (2013). DNA replication timing. Cold Spring Harb. Perspect. Biol..

[63] Boulos R.E., Drillon G., Argoul F., Arneodo A., Audit B. (2015). Structural organization of human replication timing domains. FEBS Lett..

[64] Foti R., Gnan S., Cornacchia D., Dileep V., Bulut-Karslioglu A., Diehl S., Buness A., Klein F.A., Huber W., Johnstone E. (2016). Nuclear Architecture Organized by Rif1 Underpins the Replication-Timing Program. Mol. Cell.

[65] Julienne H., Zoufir A., Audit B., Arneodo A. (2013). Human genome replication proceeds through four chromatin states. PLoS Comput. Biol..

[66] Kolesnikova T.D. (2013). Regulation of DNA replication timing. Mol. Biol..

[67] Pliss A., Malyavantham K.S., Bhattacharya S., Berezney R. (2013). Chromatin dynamics in living cells: Identification of oscillatory motion. J. Cell. Physiol..

[68] Bornfleth H., Edelmann P., Zink D., Cremer T., Cremer C. (1999). Quantitative Motion Analysis of Subchromosomal Foci in Living Cells Using Four-Dimensional Microscopy. Biophys. J..

[69] Oomen M.E., Dekker J. (2017). Epigenetic characteristics of the mitotic chromosome in 1D and 3D. Crit. Rev. Biochem. Mol. Biol..

[70] Mirny L.A. (2011). The fractal globule as a model of chromatin architecture in the cell. Chromosom. Res..

[71] Teif V.B., Bohinc K. (2011). Condensed DNA: Condensing the concepts. Prog. Biophys. Mol. Biol..

[72] Hübner B., Cremer T., Neumann J. (2013). Correlative Microscopy of Individual Cells: Sequential Application of Microscopic Systems with Increasing Resolution to Study the Nuclear Landscape. Methods Mol. Biol..

[73] Lloyd D. (2009). Oscillations, Synchrony and Deterministic Chaos. Prog. Bot..

[74] Prigogine I. (1977). Time, Structure and Fluctuations. Nobel Lect. Chem..

[75] Prigogine I., Stengers I. (1984). Order out of Chaos: Man’s New Dialogue with Nature.

[76] Olemskoi A.I., Khomenko A.V., Olemskoi D.A. (2004). Field theory of self-organization. Phys. A Stat. Mech. Appl..

[77] Zhukotsky A.V., Butusova N.N., Shchegolev A.J., Kogan E.M. (1985). A vector model of structural regulation in cell nucleus following exposure to Phenobarbital. Biofizika.

[78] Gates R.R., Ruggles Gates R. (1942). Nucleoli and related nuclear structures. Bot. Rev..

[79] Maszewski J., Kwiatkowska M. (1984). Number, size, and transcriptional activity of nucleoli during different periods of interphase in antheridial filaments of *Chara vulgaris* L.. Folia Histochem. Cytobiol..

[80] Almeira N., Risau-Gusman S. (2017). Role of transcriptional bursts in cellular oscillations. J. Biol..

[81] Fukaya T., Lim B., Levine M. (2016). Enhancer Control of Transcriptional Bursting. Cell.

[82] Symmons O., Raj A. (2016). What’s Luck Got to Do with It: Single Cells, Multiple Fates, and Biological Nondeterminism. Mol. Cell.

[83] Wang Y., Ni T., Wang W., Liu F. (2019). Gene transcription in bursting: A unified mode for realizing accuracy and stochasticity. Biol. Rev..

[84] Erenpreisa J., Budylin A. (1990). Related changes in RNA synthesis and DNA superhelicity during starvation of Ehrlich ascites tumour cells. Proc. Latv. Acad. Sci..

[85] Lloyd D., Murray D.B. (2006). The temporal architecture of eukaryotic growth. FEBS Lett..

[86] Tsuchyia M., Wong S.T., Yeo Z.X., Colosimo A., Palumbo M.C., Farina L., Crescenzi M., Mazzola A., Negri R., Bianchi M.M. (2007). Gene expression waves. FEBS J..

[87] Lloyd D., Murray D.B. (2005). Ultradian metronome: Timekeeper for orchestration of cellular coherence. Trends Biochem. Sci..

[88] Liu S., Chen H., Ronquist S., Seaman L., Ceglia N., Meixner W., Chen P.Y., Higgins G., Baldi P., Smale S. (2018). Genome Architecture Mediates Transcriptional Control of Human Myogenic Reprogramming. iScience.

[89] Pederson T., King M.C., Marko J.F. (2015). Forces, fluctuations, and self-organization in the nucleus. Mol. Biol. Cell.

[90] Vickaryous M.K., Hall B.K. (2006). Human cell type diversity, evolution, development, and classification with special reference to cells derived from the neural crest. Biol. Rev..

[91] Cope F.O., Willie J.J. (1991). Carcinogenesis and Apoptosis: Paradigms and paradoxes in cell cycle and differentiation. Apoptosis: The Molecular Basis of Cell Death.

[92] Lorenz E.N. (1963). Deterministic Nonperiodic Flow. J. Atmos. Sci..

[93] Erenpreisa J., Roach H.I. (1996). Epigenetic selection as a possible component of transdifferentiation. Further study of the commitment of hypertrophic chondrocytes to become osteocytes. Mech. Ageing Dev..

[94] Noble R., Noble D. (2018). Harnessing stochasticity: How do organisms make choices?. Chaos.

[95] Wapenaar K., Snieder R. (2007). Determinism: Chaos tamed. Nature.

[96] Erenpreisa J. (2000). “Tamed” chaos in embryonal development and carcinogenesis: A holistic view. Proc. Latv. Acad. Sci. Sect. B.

[97] Mojtahedi M., Skupin A., Zhou J., Castaño I.G., Leong-Quong R.Y.Y., Chang H., Trachana K., Giuliani A., Huang S. (2016). Cell Fate Decision as High-Dimensional Critical State Transition. PLoS Biol..

[98] Chang H.H., Hemberg M., Barahona M., Ingber D.E., Huang S. (2008). Transcriptome-wide noise controls lineage choice in mammalian progenitor cells. Nature.

[99] Ben-Porath I., Thomson M.W., Carey V.J., Ge R., Bell G.W., Regev A., Weinberg R.A. (2008). An embryonic stem cell-like gene expression signature in poorly differentiated aggressive human tumors. Nat. Genet..

[100] Erenpreisa J., Salmina K., Huna A., Jackson T.R., Vazquez-Martin A., Cragg M.S. (2015). The “virgin birth”, polyploidy, and the origin of cancer. Oncoscience.

[101] Erenpreiss J.O. (1993). Current Concepts of Malignant Growth.

[102] Illmensee K., Mintz B. (1976). Totipotency and normal differentiation of single teratocarcinoma cells cloned by injection into blastocysts. Proc. Natl. Acad. Sci. USA.

[103] Kim A., Cohen M.S. (2016). The discovery of vemurafenib for the treatment of BRAF-mutated metastatic melanoma. Expert Opin. Drug Discov..

[104] Kleinsmith L.J., Pierce G.B. (1964). Multipotentiality of single embryonal carcinoma cells. Cancer Res..

[105] Pierce G.B., Barry Pierce G. (1985). Carcinoma is to Embryology as Mutation is to Genetics. Am. Zool..

[106] Pisco A.O., Huang S. (2015). Non-genetic cancer cell plasticity and therapy-induced stemness in tumour relapse: “What does not kill me strengthens me”. Br. J. Cancer.

[107] Salmina K., Gerashchenko B.I., Hausmann M., Vainshelbaum N.M., Zayakin P., Erenpreiss J., Freivalds T., Cragg M., Erenpreisa J. (2019). When Three Isn’t a Crowd: A Digyny Concept for Treatment-Resistant, Near-Triploid Human Cancers. Genes.

[108] Shaffer S.M., Dunagin M.C., Torborg S.R., Torre E.A., Emert B., Krepler C., Beqiri M., Sproesser K., Brafford P.A., Xiao M. (2017). Rare cell variability and drug-induced reprogramming as a mode of cancer drug resistance. Nature.

[109] Stevens L.C. (1964). Experimental production of testicular teratomas in mice. Proc. Natl. Acad. Sci. USA.

[110] Vainshelbaum N.M., Zayakin P., Kleina R., Giuliani A., Erenpreisa J. (2019). Meta-Analysis of Cancer Triploidy: Rearrangements of Genome Complements in Male Human Tumors Are Characterized by XXY Karyotypes. Genes.

[111] Vinnitsky V.B. (1993). Oncogerminative hypothesis of tumor formation. Med. Hypotheses.

[112] Zhang S., Mercado-Uribe I., Xing Z., Sun B., Kuang J., Liu J. (2014). Generation of cancer stem-like cells through the formation of polyploid giant cancer cells. Oncogene.

[113] Niu N., Mercado-Uribe I., Liu J. (2017). Dedifferentiation into blastomere-like cancer stem cells via formation of polyploid giant cancer cells. Oncogene.

[114] Liu C., Cai Z., Jin G., Peng D., Pan B.S., Zhang X., Han F., Xu X., Lin H.K. (2018). Abnormal gametogenesis induced by p53 deficiency promotes tumor progression and drug resistance. Cell Discov..

[115] Kastan M.B. (2007). Wild-Type p53: Tumors Can’t Stand It. Cell.

[116] Meshorer E., Yellajoshula D., George E., Scambler P.J., Brown D.T., Misteli T. (2006). Hyperdynamic plasticity of chromatin proteins in pluripotent embryonic stem cells. Dev. Cell.

[117] Bernstein B.E., Mikkelsen T.S., Xie X., Kamal M., Huebert D.J., Cuff J., Fry B., Meissner A., Wernig M., Plath K. (2006). A Bivalent Chromatin Structure Marks Key Developmental Genes in Embryonic Stem Cells. Cell.

[118] Evan G., Harrington E., Fanidi A., Land H., Amati B., Bennett M. (1994). Integrated control of cell proliferation and cell death by the c-myc oncogene. Philos. Trans. R. Soc. Lond. B.

[119] Tyler A.L., Crawford D.C., Pendergrass S.A. (2014). Detecting and characterizing pleiotropy: New methods for uncovering the connection between the complexity of genomic architecture and multiple phenotypes- session introduction. Biocomputing.

[120] Baryshev M., Inashkina I., Salmina K., Huna A., Jackson T.R., Erenpreisa J. (2018). DNA methylation of the Oct4A enhancers in embryonal carcinoma cells after etoposide treatment is associated with alternative splicing and altered pluripotency in reversibly senescent cells. Cell Cycle.

[121] Erenpreisa J., Salmiņa K., Belyayev A., Inashkina I., Cragg M.S. (2017). Survival at the Brink. Autophagy: Cancer, Other Pathologies, Inflammation, Immunity, Infection, and Aging.

[122] Kossiakoff A., Sweet W.N., Seymour S.J., Biemer S.M. (2011). Systems Engineering Principles and Practice.

[123] Tun K., Menghini M., D’Andrea L., Dhar P., Tanaka H., Giuliani A. (2011). Why so Few Drug Targets: A Mathematical Explanation?. Curr. Comput..

[124] Illidge T. (2000). Polyploid giant cells provide a survival mechanism for p53 mutant cells after dna damage. Cell Biol. Int..

[125] Lagadec C., Vlashi E., Della Donna L., Dekmezian C., Pajonk F. (2012). Radiation-Induced Reprogramming of Breast Cancer Cells. Stem Cells.

[126] Salmina K., Jankevics E., Huna A., Perminov D., Radovica I., Klymenko T., Ivanov A., Jascenko E., Scherthan H., Cragg M. (2010). Up-regulation of the embryonic self-renewal network through reversible polyploidy in irradiated p53-mutant tumour cells. Exp. Cell Res..

[127] Chen J., Niu N., Zhang J., Qi L., Shen W., Donkena K.V., Feng Z., Liu J. (2019). Polyploid Giant Cancer Cells (PGCCs): The Evil Roots of Cancer. Curr. Cancer Drug Targets.

[128] Mirzayans R., Andrais B., Murray D. (2018). Roles of Polyploid/Multinucleated Giant Cancer Cells in Metastasis and Disease Relapse Following Anticancer Treatment. Cancers.

[129] Conant G.C. (2010). Rapid reorganization of the transcriptional regulatory network after genome duplication in yeast. Proc. Biol. Sci..

[130] Huang S., Ernberg I., Kauffman S. (2009). Cancer attractors: A systems view of tumors from a gene network dynamics and developmental perspective. Semin. Cell Dev. Biol..

[131] Mosieniak G., Sikora E. (2010). Polyploidy: The link between senescence and cancer. Curr. Pharm. Des..

[132] Davoli T., Denchi E.L., de Lange T. (2010). Persistent telomere damage induces bypass of mitosis and tetraploidy. Cell.

[133] Wang Q., Wu P.C., Dong D.Z., Ivanova I., Chu E., Zeliadt S., Vesselle H., Wu D.Y. (2013). Polyploidy road to therapy-induced cellular senescence and escape. Int. J. Cancer.

[134] Erenpreisa J., Salmina K., Cragg M.S., Dorszewska J., Kozubsk W. (2017). Accelerated Senescence of Cancer Stem Cells: A Failure to Thrive or a Route to Survival?. Senescence Physiology or Pathology.

[135] Erenpreisa J., Cragg M.S. (2013). Three steps to the immortality of cancer cells: Senescence, polyploidy and self-renewal. Cancer Cell Int..

[136] Vincent M. (2012). Cancer: A de-repression of a default survival program common to all cells?. BioEssays.

[137] Walther V., Hiley C.T., Shibata D., Swanton C., Turner P.E., Maley C.C. (2015). Can oncology recapitulate paleontology? Lessons from species extinctions. Nat. Rev. Clin. Oncol..

[138] Trigos A.S., Pearson R.B., Papenfuss A.T., Goode D.L. (2017). Altered interactions between unicellular and multicellular genes drive hallmarks of transformation in a diverse range of solid tumors. Proc. Natl. Acad. Sci. USA.

[139] Trigos A.S., Pearson R.B., Papenfuss A.T., Goode D.L. (2018). How the evolution of multicellularity set the stage for cancer. Br. J. Cancer.

[140] Vincent M.D. (2011). Cancer: Beyond speciation. Adv. Cancer Res..

[141] Vinogradov A.E. (2010). Human transcriptome nexuses: Basic-eukaryotic and metazoan. Genomics.

[142] Vinogradov A.E., Anatskaya O.V. (2019). Evolutionary framework of the human interactome: Unicellular and multicellular giant clusters. Biosystem.

[143] Erenpreisa J., Giuliani A., Vinogradov A.E., Anatskaya O.V., Vazquez-Martin A., Salmina K., Cragg M.S. (2018). Stress-induced polyploidy shifts somatic cells towards a pro-tumourogenic unicellular gene transcription network. Cancer Hypotheses.

[144] Vazquez-Martin A., Anatskaya O.V., Giuliani A., Erenpreisa J., Huang S., Salmina K., Inashkina I., Huna A., Nikolsky N.N., Vinogradov A.E. (2016). Somatic polyploidy is associated with the upregulation of c-MYC interacting genes and EMT-like signature. Oncotarget.

[145] Kondrashov A.S. (1997). Evolutionary genetics of life cycles. Annu. Rev. Ecol. Syst..

[146] Raikov I.B. (1982). The Protozoon Nucleus–Morphology and Evolution.

[147] Demin S.Y., Berdieva M.A., Goodkov A.V. (2019). Cyclic Polyploidy in Obligate Agamic Amoebae. Cell Tissue Biol..

[148] Bell G. (1985). The origin and early evolution of germ cells as illustrated by the Volvocales. The Origin and Evolution of Sex.

[149] Berdieva M., Demin S., Goodkov A. (2019). Amoeba proteus and ploidy cycles: From simple model to complex issues. Protistology.

[150] Maciver S.K. (2016). Asexual Amoebae Escape Muller’s Ratchet through Polyploidy. Trends Parasitol..

[151] Domazet-Lošo T., Klimovich A., Anokhin B., Anton-Erxleben F., Hamm M.J., Lange C., Bosch T.C.G. (2014). Naturally occurring tumours in the basal metazoan Hydra. Nat. Commun..

[152] Nanjundiah W., Kal N., Stuart N. (2016). Cellular slime mold development as a paradigm for the transition from Unicellular to Multicellular life. Multicellularity. Origins and Evolution.

[153] Ledbetter D.H. (2009). Chaos in the embryo. Nat. Med..

[154] Vanneste E., Voet T., Le Caignec C., Ampe M., Konings P., Melotte C., Debrock S., Amyere M., Vikkula M., Schuit F. (2009). Chromosome instability is common in human cleavage-stage embryos. Nat. Med..

[155] Peaston A.E., Knowles B.B., Hutchison K.W. (2007). Genome plasticity in the mouse oocyte and early embryo. Biochem. Soc. Trans..

[156] Zernicka-Goetz M., Huang S. (2010). Stochasticity versus determinism in development: A false dichotomy?. Nat. Rev. Genet..

[157] Lotem J., Sachs L. (2002). Epigenetics wins over genetics: Induction of differentiation in tumor cells. Semin. Cancer Biol..

[158] Erenpreisa J. (2019). Janis Olgerts Erenpreiss and his school of cancer research: Commemorating the 90th anniversary. Proc. Latv. Acad. Sci. Sect. B.

[159] Bissell M.J., Radisky D. (2001). Putting tumours in context. Nat. Rev. Cancer.

[160] Teif V.B., Mallm J.-P., Sharma T., Mark Welch D.B., Rippe K., Eils R., Langowski J., Olins A.L., Olins D.E. (2017). Nucleosome repositioning during differentiation of a human myeloid leukemia cell line. Nucleus.

[161] Amson R., Karp J.E., Telerman A. (2013). Lessons from tumor reversion for cancer treatment. Curr. Opin. Oncol..

[162] Lipkin G. (2008). Plasticity of the cancer cell: Implications for epigenetic control of melanoma and other malignancies. J. Investig. Dermayol..

[163] Kauffman S. (1971). Differentiation of malignant to benign cells. J. Theor. Biol..

[164] Huang S. (2004). Back to the biology in systems biology: What can we learn from biomolecular networks?. Brief. Funct. Genom. Proteom..

[165] Li Q., Wennborg A., Aurell E., Dekel E., Zou J.-Z., Xu Y., Huang S., Ernberg I. (2016). Dynamics inside the cancer cell attractor reveal cell heterogeneity, limits of stability, and escape. Proc. Natl. Acad. Sci. USA.

[166] Pinto M.C.X., Tonelli F.M.P., Vieira A.L.G., Kihara A.H., Ulrich H., Resende R.R. (2016). Studying complex system: Calcium oscillations as attractor of cell differentiation. Integr. Biol..

[167] Takahashi K., Yamanaka S. (2006). Induction of pluripotent stem cells from mouse embryonic and adult fibroblast cultures by defined factors. Cell.

[168] Strickland S., Mahdavi V. (1978). The induction of differentiation in teratocarcinoma stem cells by retinoic acid. Cell.

[169] Breitman T.R., Selonick S.E., Collins S.J. (1980). Induction of differentiation of the human promyelocytic leukemia cell line (HL-60) by retinoic acid. Proc. Natl. Acad. Sci. USA.

[170] Li Y., Agrawal I., Gong Z. (2019). Reversion of tumor hepatocytes to normal hepatocytes during liver tumor regression in an oncogene-expressing transgenic zebrafish model. Dis. Models Mech..

[171] Ishay-Ronen D., Christofori G. (2019). Targeting Cancer Cell Metastasis by Converting Cancer Cells into Fat. Cancer Res..

[172] Weaver W. (1948). Science and complexity. Am. Sci..

